# Speech Enhancement by Multiple Propagation through the Same Neural Network

**DOI:** 10.3390/s22072440

**Published:** 2022-03-22

**Authors:** Tomasz Grzywalski, Szymon Drgas

**Affiliations:** Institute of Automatic Control and Robotics, Poznan University of Technology, 60-965 Poznan, Poland; grzywalski@stethome.com

**Keywords:** speech, enhancement, multi-pass, U-Net, ResBLSTM, Transformer-Net

## Abstract

Monaural speech enhancement aims to remove background noise from an audio recording containing speech in order to improve its clarity and intelligibility. Currently, the most successful solutions for speech enhancement use deep neural networks. In a typical setting, such neural networks process the noisy input signal once and produces a single enhanced signal. However, it was recently shown that a U-Net-based network can be trained in such a way that allows it to process the same input signal multiple times in order to enhance the speech even further. Unfortunately, this was tested only for two-iteration enhancement. In the current research, we extend previous efforts and demonstrate how the multi-forward-pass speech enhancement can be successfully applied to other architectures, namely the ResBLSTM and Transformer-Net. Moreover, we test the three architectures with up to five iterations, thus identifying the method’s limit in terms of performance gain. In our experiments, we used the audio samples from the WSJ0, Noisex-92, and DCASE datasets and measured speech enhancement quality using SI-SDR, STOI, and PESQ. The results show that performing speech enhancement up to five times still brings improvements to speech intelligibility, but the gain becomes smaller with each iteration. Nevertheless, performing five iterations instead of two gives additional a 0.6 dB SI-SDR and four-percentage-point STOI gain. However, these increments are not equal between different architectures, and the U-Net and Transformer-Net benefit more from multi-forward pass compared to ResBLSTM.

## 1. Introduction

Speech enhancement allows for the extraction of the desired speech signal from a mixture of speech with interfering sounds coming from different sources. These methods can be used in hearing aids [1], smartphones [2], or, as a pre-processing step, in automatic speech or speaker recognition [3].

In recent years, great progress has been made with deep neural networks (DNNs) applied to speech enhancement. In these methods, a degraded signal is provided with the input of the neural network, which is trained to provide an estimate of the clean, undistorted signal. The representation of a signal at the input of the DNN can be a spectrogram, MFCCs (mel-frequency cepstral coefficients), or raw (time-domain) signal. The network can be trained to obtain a mask (e.g., an IRM (ideal ratio mask, [4]), a cIRM (a complex ideal ratio mask, [5])), or a representation from which the signal can be reconstructed (for example, the STFT (short-term Fourier transform) of the clean speech signal).

There are many neural network architectures for speech enhancement that can be found in the literature. These include DNNs with fully-connected layers [6,7], fully-convolutional networks [8], recurrent neural networks (RNNs) [9], and combinations of convolutional and recurrent neural networks [10]. Convolutional layers used in speech-enhancement neural networks often use a dilation greater than one, which helps in utilizing long-range dependencies without increasing the number of models’ parameters [11,12,13].

An interesting example of RNNs for speech enhancement is ResBLSTM [14,15], which uses a skip connection between the input to LSTM (long short-term memory) and its output. Such a connection results in a performance boost in comparison to pure stacked LSTM layers. The presence of such a connection forces the layer to learn residual mapping, and it can potentially take long context into account.

Recently, a multi-head attention [16] has been used in speech enhancement [17,18,19]. This technique provides an alternative way to take into account possibly large contexts of the input signal. Neural models that use self-attention are, by convention, named transformer networks. Currently, a lot of research is devoted to optimizing ways of encoding positional information in sequences on the input to the transformer networks. Except for the absolute positional encoding used in the original research paper [16], relative [20] and continuous dynamic [21] positional encodings have also been proposed, but their performance in speech enhancement has not yet been tested.

One of the neural network architectures used in this work comes from our earlier work on U-Net-based speech enhancement [22,23]. The results presented in these works suggest that when the time-frequency resolution of hidden activations (feature maps) is not reduced by max-pooling or strided convolutions, a better performance can be achieved in terms of SDR (signal-to-distortion ratio), STOI (spectro-temporal objective intelligibility), and PESQ (perceptual evaluation of speech quality). However, to achieve this performance, the receptive field needs to be enlarged using dilated convolutions or recurrent layers.

Although there has been progress in DNN-based speech enhancement, the enhanced speech is still not perfect and may not be intelligible. When this occurs, it is mainly due to the existence of the residual noise and distortions of the speech. In the case of residual noise, one can imagine that processing the noisy signal many times with the same network can bring the desired effect. However, the accumulation of distortions and loss of the speech information can make this task difficult.

A recent technique to improve performance of neural networks is progressive learning. In the context of speech enhancement, progressive learning was applied by sequentially connecting many sub-networks with shared weights [24]. The subsequent networks had different SNRs of target representations. The target SNRs in the subsequent sub-networks were from the lowest (slightly enhanced speech) to the highest (clean speech). As such, each sub-network removed a portion of the noise present in the noisy input signal, hence the name “progressive”.

In this paper, multi-pass speech enhancement is proposed. Using this method, in the original neural network architecture (which can be any one of the most popular neural networks used for speech enhancement), three main blocks are identified: input, base, and output. The output from the input block is processed by the same base block many times. Finally, the outputs are computed using the output block. We test this scheme for the following neural network architectures: (1) U-Net with dilated convolutions, (2) RNN, and (3) Transformer. The improvement in speech enhancement after subsequent passes through the base block is achieved thanks to a special skip connection from the previous pass, as in [25].

The main difference between the method proposed in this paper and the progressive learning described in [24,26,27] is that the original noisy signal is provided at the input of each step, while the target at the output of each step is the clean signal. In this regard, instead of removing a certain portion of the input noise, our proposed solution attempts to remove all the noise at each processing step, but with each additional step, this process becomes more refined.

The paper is structured as follows. In Section 2, the undertaken problem is formulated, the proposed multi-pass approach is introduced, and the datasets and experimental setup are described. In Section 3, the results are presented and analyzed. Next, the discussion of the results in the context of the literature is presented in Section 4. Finally, the conclusions are enumerated in Section 5.

## 2. Materials and Methods

### 2.1. Problem Formulation

We consider the speech enhancement problem in which a speech signal is corrupted by an additive noise, i.e.,

(1) y(k)=x(k)+d(k),

where y(k), x(k), and d(k) are the *k*′th samples of the mixture, clean speech, and noise, respectively.

In order to attenuate the noise, a neural network can be employed. In this article, we consider networks that accept on input a tensor $Y\in R^{N\times B\times T\times 2}$ that contains STFT of the noisy speech, where the first dimension denotes an example in the batch with *N* examples, *B* and *T* denote the number of frequency bands and the number of frames, respectively, and the last dimension refers to real and imaginary part of the STFT. The neural network maps this input tensor to the output tensor $O\in R^{N\times B\times T\times 2}$, which should predict $M\in R^{N\times B\times T\times 2}$ containing cIRM (processed by a nonlinear function to limit the range of values; see Section 2.6 for more details), which is further used to compute the STFT of the estimated clean speech signal.

In this article, the speech enhancement neural network is built from three main subnetworks: input layers, base layers, and output layers. The input subnetwork extracts a representation of the input mixture. Next, this representation is processed many times by the main subnetwork’s base layers, which refine the representation in such a way that the output subnetwork can project these representations to cIRM masks, which will provide a high-quality speech signal.

### 2.2. Multi-Pass

**Figure 1:**
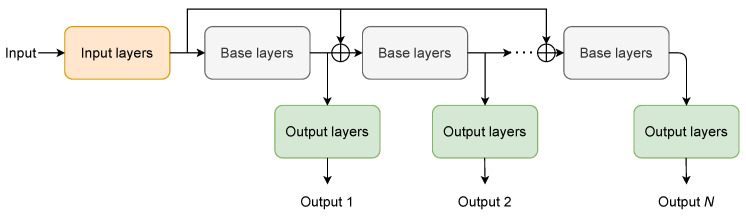
The general architecture of multi-pass speech enhancement. The ⊕ symbol represents element-wise addition operation.

The general processing scheme used in multi-pass speech enhancement is shown in Figure 1. The input tensor is processed by input layers to extract a representation of the input signal. Next, this representation is processed by the base subnetwork *L* times. There are skip connections that add the feature representation of the input signal to the output of the base subnetwork before each iteration. The output of the base subnetwork after each iteration is processed by the output layers that project the representation to the cIRM mask, $O_{l}$ for l=1,…,L, which is further used to reconstruct the estimated signal (see Section 2.5 for more information).

During the training for each output, its loss is computed as

(2) $$L_{l}=\frac{1}{2NBT}\sum_{n=1}^{N}\sum_{b=1}^{B}\sum_{t=1}^{T}\sum_{p=1}^{2}{\left({\left(O_{l}\right)}_{nbtp}-M_{nbtp}\right)}^{2}\;.$$

The total loss used to compute gradient is the sum of the losses from all outputs divided by the number of outputs

(3) $$L=\frac{1}{L}\sum_{l=1}^{L}L_{l}\;.$$

Such a combined loss function allows the trained network to process the input signal multiple times. During the second, third, and each additional iteration, the network can make use of the previously-denoised signal representation as well as the original signal representation, which allows it to enhance the signal further. The skip connections that add the feature representation of the input signal to the output from base layers before each consecutive iteration are a critical part of the proposed solution, as they prevent the accumulation of the distortion added by base layers at each pass.

In the proposed scheme, unlike in the progressive learning, each enhancement step is performed by the same set of layers and weights, which are trained to provide the best possible output signal after each pass. During inference, the multi-pass network can be used to perform any number of enhancement iterations between 1 and *L*. In that case, only the final output should be used, as it contains the most refined cIRM mask. In progressive learning, each processing stage is realized by a separate network that removes a certain, pre-defined amount of noise.

### 2.3. Base Sub-Networks

The multi-pass speech enhancement can be applied on top of any existing neural network architecture. The only requirement is the distinction of the input layers, base layers, and output layers within the neural network. The best choice for input and output layers is the usage of single convolutional layers in each case. The output feature tensor from input layers needs to have the same shape as the input tensor to the output layers; otherwise, the skip connection between enhancement iteration will not be possible. If no suitable layers can be identified within the existing architecture, they can be added. In this work, we used this approach with three popular neural network designs.

#### 2.3.1. Dilated U-Net

This is a U-Net-based architecture similar to the one used in [25]. The Dilated U-Net uses two-dimensional convolutional operations to map the spectrogram representation of the noisy signal into the output cIRM mask. The base subnetwork includes three levels of convolutional layers, each separated by a skip connection that connects the encoder to the decoder. Each convolutional layer consists of 54 convolutional filters of size 3 × 3.

The feature maps at the input to each aforementioned convolutional layer are extended with features produced by processing blocks called CEMs (context extension modules). The role of CEM is to provide wide context in terms of either time or frequency without reducing the time-frequency resolution between the subsequent levels of the U-Net. This is achieved with dilated convolutions. Each CEM consists of two parallel colvolutional layers, each using eight filters and a different dilation rate. The outputs of the two layers are concatenated at the output of each CEM. The variant of CEM that extends the context in time dimension uses kernels of size (1, 7) and dilation rates of (1, 3) and (1, 4). The frequency-dilated CEMs use (7, 1) kernels and (2, 1) and (3, 1) dilation rates.

The number of filters used by the “main” convolutional filters of the U-Net (54 filters) and the number of filters inside the CEMs (2×8 filters) have been established using the same procedure as described in [25] given the context of current experiments, which essentially means that the highest possible values were used that allowed it to perform the training on a GPU with 11 GB of RAM.

**Figure 2:**
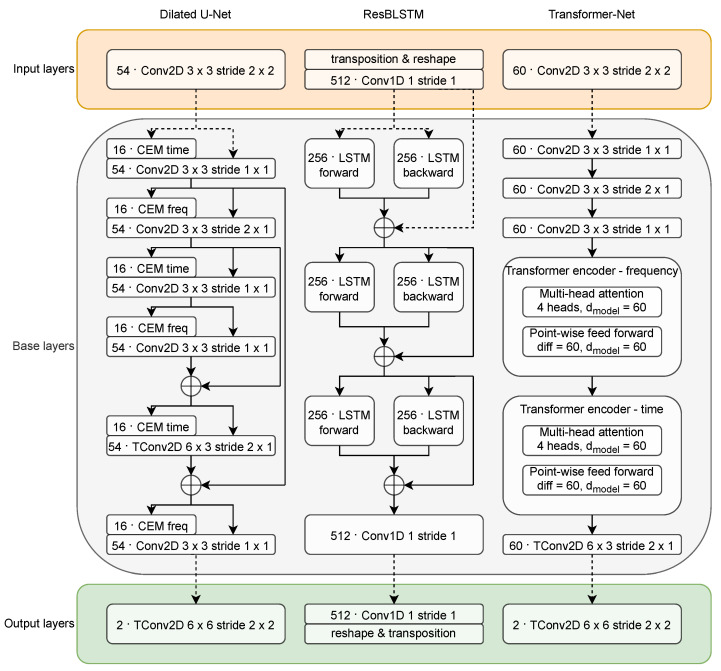
Input, Base, and Output layers in the three neural network architectures tested with multi-pass speech enhancement.

In the case of Dilated U-Net, the input layers (as defined in Figure 1) include a single convolutional layer with 54 filters with 3 × 3 kernels and stride equal to two in both time and frequency dimensions. Analogically, the output layers include a single transposed convolution with two filters (one for the real and one for the imaginary part), kernels of size 6 × 6, and stride equal to two in both dimensions. For an overview of this and other architectures, please refer to Figure 2. Please note that the first number in each block denotes the number of filters or recurrent units in case or recurrent layers.

#### 2.3.2. ResBLSTM

The ResBLSTM differs significantly from the aforementioned U-Net in terms of feature tensor composition. This base subnetwork does not distinguish between the channel dimension of the feature tensor and the frequency dimension (i.e., features at the output of any given layer in the ResBLSTM are not localized in frequency). The features at the input of this base subnetwork are organized in a three-dimensional array $F\in R^{N\times C\times T}$; i.e., for each spectrogram time frame, there are *C* features. Each of these features potentially depends on all signal’s frequencies. The recurrent layers from the ResBLSTM network iterate over time frames accepting the *C* features on the input at each step. The output layers transform this signal representation into an array, which can be reshaped and transposed into the output tensor that contains the cIRM mask.

A detailed structure of the multi-pass-enabled ResBLSTM is depicted in Figure 2. In order to fit the original noisy speech spectrogram into the three-dimensional tensor, the ResBLSTM input layers start with transposition and reshape from N×B×T×2 into N×2B×T. This tensor is then processed by a single one-dimensional convolutional layer with 512 filters of kernel size one and stride equal to one. This produces a feature tensor of shape N×C×T with C=512 that is further processed by the base subnetwork. The output layers use convolution with a kernel size of one and the number of filters is double the number of frequency bands (one set for the real and one for the imaginary part) to produce output tensor of shape N×2B×T. This is followed by the reverse reshape and transposition that restores the original tensor shape (N×B×T×2).

The ResBLSTM base subnetwork is composed of three bidirectional LSTM [28] layers with 256 units in each direction, concatenated on output and encircled with the residual connection. This module concludes with a single convolutional layer with 512 filters with kernel of size one followed by batch normalization and ELU (exponential linear unit) nonlinearity [29] defined as

(4) $$\varphi \left(x\right)=\left\{\begin{array}{cc} x\; & x>0\\ e^{x}-1\; & x\leq 0 \end{array}\right.\;.$$

Using this configuration, the ResBLSTM can be trained to transform features into a representation from which cIRM mask can be obtained. Using the capacity of a LSTM layer to store information for many recurrent steps, the ResBLSTM network can compute elements of the cIRM mask based on potentially large context, which makes it a very effective model for speech enhancement.

#### 2.3.3. Transformer-Net

Transformer networks are another powerful tools that can be employed to perform speech enhancement. Unlike the recurrent layers, they use the self-attention monarchism that can model relation between any two locations in the input signal independent on their distance. This allows the model to make use of potentially large context, which, as was previously shown in [22], is crucial for high quality speech enhancement. When working with spectrogram representations of signals, two types of distances should be considered: distance in time and frequency. To properly model each of them, two attention modules are used - one for each dimension.

Because the Transformer-Net distinguishes frequency dimension as being separate from the channel dimension, the input and output layers are configured identically as in the case of Dilated U-Net. The Transformer-Net base subnetwork starts with a sequence of three convolutional layers, each with K=60 filters with 3 × 3 kernels. Each layer ends with batch normalization and ELU nonlinearity and additionally the second layer uses stride equal two in frequency dimension (analogically as on the second level of the Dilated U-Net). This is coupled with transposed convolution with *K* filters of size 6 × 3 and stride equal two in frequency dimension, batch normalization and ELU on the output on this base subnetwork.

The central part of this base subnetwork is composed of a sequence of two transformer encoders, the first modeling features in frequency dimension and the second one in time dimension. Each of the two encoders uses a multi-head attention block [16] with four heads and $d_{model}=K$ (i.e., 15 per head). Each multi-head attention block is followed by a point-wise feed-forward network (i.e., features for each time-frequency location are transformed using the feed-forward network independently), each such feed-forward network is composed of two fully-connected layers with *K* neurons and ELU nonlinearities. For performance reasons the transformers lack the normalization layers after each multi-head attention and point-wise feed-forward networks. The value of *K*, similarly as in the case of Dilated U-Net, was set experimentally to fill the available GPU memory.

### 2.4. Dataset

The effectiveness of multi-pass speech enhancement was tested on the popular Wall Street Journal (WSJ0) dataset [30], from which only the utterances included on the SI-84 list were used. From the 83 speakers included on the list, 3 random male and 3 random female speakers were held-out for testing. This test set included 599 speech utterances. The remaining 77 speakers contributing 6637 utterances were used for training and validation (i.e., control of the model over-fitting). As can be seen, there was no overlap between the test and the training datasets.

The WSJ0 training speech utterances were mixed with a diverse set of noises coming from the FreeField72k dataset, which was also used in [25]. Motivated by the popularity of the freefield1010 dataset [31], which was also included in the DNS challenge [32], the FreeField72k extends its predecessor by taking a much bigger sample of the free field recordings from the source database. In the process of creation of the FreeField72k, the Freesound online database [33] was queried, and all of the recordings tagged with the “field-recording” tag were downloaded. For the sake of effectiveness, all recordings shorter than 10 s and those whose download time exceeded 30 s were skipped. A resulting set of 24,237 recordings was resampled to 8k samples per second and segmented into 10 s non-overlapping fragments with the restriction that no more than five fragments could be taken from one recording. This process yielded 93,301 10 s noise fragments, from which 23% were removed due to low signal level. Finally, the FreeField72k included 72,028 10 s audio excerpts coming from 19,990 original Freesound database recordings and totaled to 200 h of diverse, real-life noise.

In the test phase, the trained models were fed with WSJ0 test speech utterances mixed with babble noise (Noisex-92, [34]) and shopping mall noise (DCASE, [35]).

### 2.5. Features

In all experiments, the neural network input consisted of a scaled STFT of the speech–noise mixture sampled at 8k samples per second. The STFT was obtained by dividing the original signals into 25 ms frames with hop-time of 10 ms and applying the Hanning window. For each frame, a 512-point fast Fourier transform was derived. The resulting STFT was eventually divided by a constant (40.0) in order to reduce the range on values on the input to the neural network to roughly fit the range −1.0 to 1.0, which is known to speed up the training process.

During the inference, The cIRM mask predicted by the neural network was applied to the STFT of the mixture (complex multiplication). Finally, after multiplication by 40.0 (inverse scaling), the inverse STFT was performed in order to obtain the time-domain-enhanced signal.

### 2.6. Targets

All networks were trained to predict the cIRM mask. In order to limit the range of values in cIRM masks, a compression using hyperbolic tangent was used. The slope parameter of this compressing function was set to 0.1, which effectively limited the targets’ values to the range of (−10, 10). During the inference, the predicted mask was uncompressed using the inverse of this compressing function.

### 2.7. Hyperparameters and Training

Each neural network was trained for 400 epochs, and during each epoch, 745 model updates were performed. First, the dataset of 6637 training speech utterances was split into actual training samples (90%—5974 samples) and validation samples (10%—663 samples). During one training epoch, each of the 5974 training speech utterances was used once (but in random order). From each training speech utterances, a random 5 s excerpt was extracted and mixed with a random 5 s excerpt from the FreeField72k dataset at SNR randomly chosen from [−5, −4, −3, −2, −1, 0] dB. If a training speech utterance was shorter than 5 s, it was padded on both sides with zeros, and this padded area was masked-out from the loss computation. For each speech–noise mixture, a training target based on a cIRM mask was computed. Training examples comprising the speech–noise mixtures and a target compressed cIRMs were grouped in batches of eight and used for training. The random number generators used in the experiments were initialized with fixed seeds so that the training data sequence was identical in all experiments.

The training used the Adam optimizer [36] with an initial learning rate of 0.002, which was further reduced by 1% after each epoch.

After each training epoch, a validation was carried out that used all of the 663 validation speech samples mixed with a random (but repeatable) sequence of FreeField72k samples at 0 dB. If a new best value of SI-SDR was observed on these validation data, a new model snapshot was taken. In the case of multi-forward experiments, only the final output from the network ($O_{L}$) was used to compute the SI-SDR. The last model snapshot taken during each training was evaluated on the 599 test speech utterances mixed with different noises as described in next section.

### 2.8. Experiments

To evaluate the performance of the multi-forward speech enhancement, six models were trained. Each of the three architectures described in Section 2.3 was trained two times: first with L=1, which is the baseline approach that performs the speech enhancement once, and with L=5, which is when the network is trained to perform the speech enhancement up to five times.

Models trained with L=1 were tested with only one speech enhancement iteration (using its only output). The model trained with L=5 was tested on all five outputs, i.e., when performing 1, 2, 3, 4, and 5 enhancement passes ($O_{1},O_{2},\ldots ,O_{5}$). In each case, the models were tested for removal of babble noise from Noisex-92 and shopping mall noise from DCASE at −5, 0, 5, and 10 dB SNR. The effectiveness of the enhancement was evaluated using SI-SDR (scale-independent SDR), STOI, and PESQ metrics.

## 3. Results

**Figure 3:**
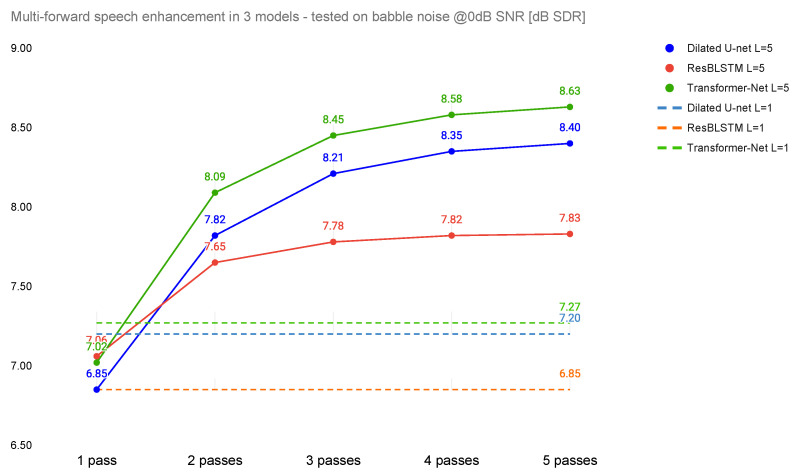
Dependence of SI-SDR on number of passes.

**Figure 4:**
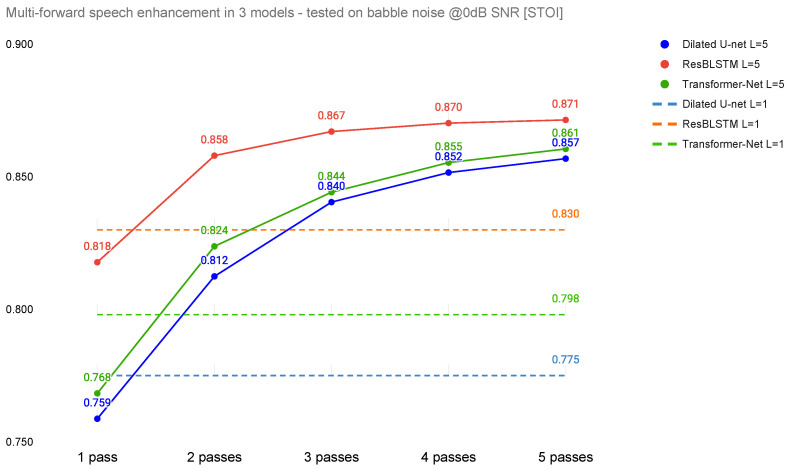
Dependence of STOI on number of passes.

The performance of the proposed method (in terms of SI-SDR and STOI) for babble noise at 0dB SNR is shown in Figure 3 and Figure 4. The horizontal dashed lines represent the performance of the models trained with L=1 and therefore serve as baselines for the three base architectures (Dilated U-Net, ResBLSTM, and Transformer-Net) trained in the multi-pass manner. In the case of the SI-SDR metric, it can be noticed that the multi-pass approach outperformed baselines by 1.36 dB for Transformer-Net, 1.2 dB for Dilated U-Net, and 0.98 dB for ResBLSTM. In the case of STOI, the improvements are: 0.041 for ResBLSTM, 0.063 for Transformer-Net, and finally 0.082 for Dilated U-Net.

When the Dilated U-Net and Transformer-Net architectures are trained in a multi-pass manner (L=5), their performance for one-pass denoising during the test (for output $O_{1}$) is worse than for the same network trained using single-pass (L=1). This is not always true for ResBLSTM, where the SI-SDR for (L=5 and output $O_{1}$) is higher than for the baseline model (L=1, $O_{1}$).

The highest difference in performance of the multi-pass approach is between the first and the second pass. With additional passes, the benefit decreases. The highest difference between four and five passes is achieved with Transformer-Net (0.05 dB SI-SDR and 0.006 STOI), while the lowest is achieved for ResBLSTM (0.01 dB SI-SDR and 0.001 STOI).

In terms of SI-SDR, the best-performing system is Transformer-Net, with five passes (SDR = 8.63 dB). However, this is not the case for the STOI metric, where the best performance was achieved using ResBLSTM (STOI = 0.871).

The results are presented in Table 1 and Table 2. They contain evaluation metrics obtained for babble noise and shopping mall noise, respectively, for all tested conditions (SNR −5, 0, 5, and 10 dB) and all metrics (SI-SDR, STOI, and PESQ).

The multi-pass approach gave substantial performance gain for all tested conditions. In the case of babble noise, the best results in terms of SI-SDR were achieved by Transformer-Net, while in terms of STOI, the best results were achieved by ResBLSTM (at lower SNR values) and Transformer-Net (at higher SNR values). In the case of shopping mall noise, Transformer-Net was the best in terms of SI-SDR (at all SNR values) and PESQ (at higher SNR values), while in terms of STOI, Dilated U-Net gave the best outcome.

It can be also noticed that ResBLSTM can bring some drop in performance between four and five passes for the PESQ metric. This occurred for both babble and shopping mall noise.

**Figure 5:**
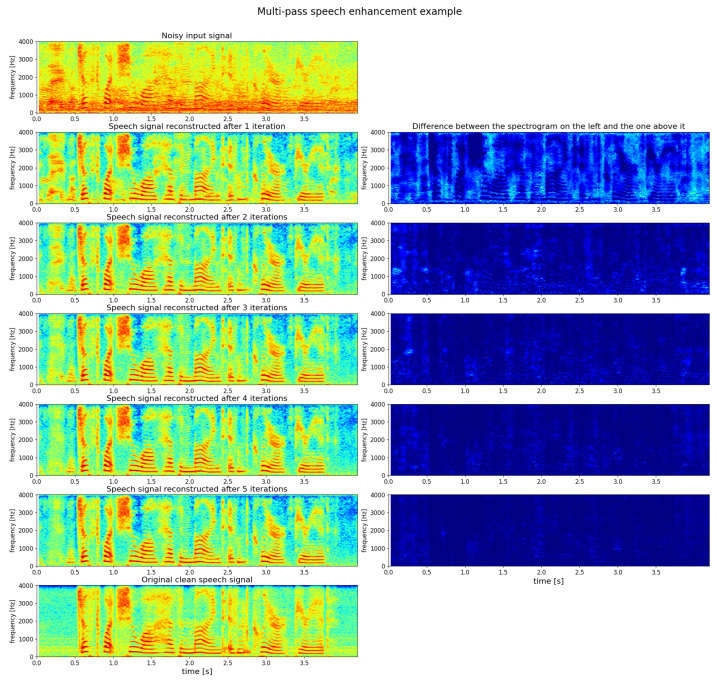
Example of multi-forward speech enhancement (Transformer-Net, babble noise at 0 dB SNR). Warmer colors indicate higher energy in given time frame and frequency band.

An example of multi-forward speech enhancement performed by Transformer-Net is provided in Figure 5. On the top left, the magnitude spectrogram of a noisy speech utterance is presented. Below it, there are the spectrograms of the denoised signals obtained after subsequent passes through the Transformer-Net (O=1, 2, 3, 4, and 5). Furthermore, finally, at the bottom, the clean (target) utterance is presented. At the right-hand side of each spectrogram of reconstructed clean speech signal, there is an image showing the difference between this spectrogram and the spectrogram located directly above it. As such, these right-hand-side images show the additional amount of noise removed at each pass. It can be noticed that with subsequent passes, residual noise patterns (concentrated in time and frequency) are removed.

## 4. Discussion

The presented results show a very clear advantage of adding the multi-pass functionality to the existing well-established neural network solutions for speech enhancement. Even if the starting architecture does not contain suitable candidate layers for input and output sub-networks as described in Figure 1, it is enough to add one or two convolutional layers at the beginning and/or end of the network to serve this purpose.

The only true disadvantage of the proposed solution that we were able to identify is the additional requirement for GPU memory during training. When using the loss function proposed in this work, and under the assumption that the base layers from Figure 1 contain the vast majority of the network’s layers, the requirement to train a multi-pass network for *L* passes in almost *L* times as high as for the baseline network that is trained to do a single enhancement pass. Some possible solutions were presented in [25], but they do not seem to scale well with values of *L* higher than three. However, the memory consumption during inference is identical as for the baseline model, and obviously the processing time scales linearly with the number of passes.

Amongst the three tested neural network architectures, this was a considerable factor for the Dilated U-Net and the Transformer-Net, which use feature tensors with two spatial dimensions: time and frequency. Because these networks preserve the feature’s location in the frequency dimension, their memory consumption is significantly higher, so the additional memory required to train these models in multi-pass manner should be taken into consideration. This is a much smaller issue in the case of the ResBLSTM, where features are located only in time, and therefore they are much more compact.

Finally, we would like to highlight two areas where the proposed solution might be especially useful. The first application is related to mobile devices, where the network sizes are relatively small. In this case, the training is usually performed on separate machines with abundant resources, so increasing the amount of memory used during training should not be an issue. However, the big advantage of the proposed solution is that it yields a potentially small, flexible model that is able to perform speech enhancement of varying quality, and the quality scales logarithmically with the number of passes. We can therefore deliver the same model to the smaller/older-generation devices, which will perform one-pass enhancement and provide a well-enhanced signal, and to the more powerful devices which, can perform multiple passes in the same time window to benefit from the increased speech denoising quality.

The second application that might significantly benefit from the proposed solution is the high-throughput network services that usually deal with varying load. Because the multi-pass networks are flexible, the number of speech enhancement iterations can be dynamically adjusted depending on the current utilization of server resources. For example, if the load is low, all five iterations might be performed. However, when the load increases, the number of iterations might be dynamically reduced so that the service is able to process all requests on time. Such a solution should result in a more effective resource utilization and improve the end-user experience.

## 5. Conclusions

A deepened research of the multi-pass enhancement methods has revealed that this technique is applicable to a wide range of machine learning models, and in each case it brings significant improvement in the speech enhancement performance as measured by SI-SDR, STOI, and PESQ. As expected, with each additional iteration, the increments in performance become smaller, but it is still reasonable to perform five passes, and additional ones should improve the signal quality even further. The proposed solution is quite simple in theory and implementation and should be especially useful with smaller models that have limited hardware resources available during inference or to optimize resource utilization by network services that perform online speech enhancement.

## Figures and Tables

**Table 1:** Babble noise (TIMIT). Best performances in each category are marked with bold font.

Metric	*L*	*O*	SI-SDR (dB)				STOI				PESQ			
SNR			−5 dB	0 dB	5 dB	10 dB	−5 dB	0 dB	5 dB	10 dB	−5 dB	0 dB	5 dB	10 dB
original	-	-	−5.04	−0.04	4.96	9.97	0.375	0.555	0.716	0.833	1.67	1.92	2.23	2.55
Dilated	1	1	1.82	7.20	11.21	13.70	0.575	0.775	0.894	0.948	1.90	2.41	2.80	3.10
U-Net	5	1	1.41	6.85	10.84	12.89	0.559	0.759	0.882	0.940	1.85	2.35	2.75	3.05
		2	2.51	7.82	11.75	14.44	0.612	0.812	0.917	0.959	1.97	2.48	2.86	3.17
		3	3.01	8.21	11.99	14.80	0.644	0.840	0.930	0.964	2.03	2.54	2.92	3.21
		4	3.26	8.35	12.06	14.91	0.658	0.852	0.934	0.966	2.06	2.57	2.94	3.22
		5	3.37	8.40	12.06	14.95	0.667	0.857	0.935	0.966	2.07	2.58	2.95	3.22
Res	1	1	2.16	6.85	10.53	13.30	0.634	0.830	0.919	0.956	1.98	2.50	2.90	3.21
BLSTM	5	1	2.12	7.06	10.81	13.60	0.623	0.818	0.919	0.959	1.93	2.45	2.88	3.21
		2	2.92	7.65	11.28	14.09	0.668	0.858	0.934	0.963	2.05	2.58	2.98	3.28
		3	3.15	7.78	11.37	14.14	0.686	0.867	0.936	0.964	2.07	2.60	3.00	3.29
		4	3.22	7.82	11.39	14.13	0.692	0.870	0.937	0.964	2.08	2.61	3.00	3.29
		5	3.25	7.83	11.39	14.12	**0.696**	**0.871**	0.937	0.964	2.08	2.60	3.00	3.28
Trans-	1	1	2.06	7.27	11.35	14.37	0.598	0.798	0.909	0.957	1.87	2.38	2.79	3.12
former	5	1	1.63	7.02	11.13	13.87	0.567	0.768	0.888	0.946	1.87	2.37	2.78	3.10
-Net		2	2.82	8.09	12.08	15.24	0.625	0.824	0.922	0.962	2.01	2.52	2.92	3.23
		3	3.49	8.45	12.34	15.55	0.656	0.844	0.932	0.966	2.09	2.60	2.99	3.28
		4	3.71	8.58	12.41	15.62	0.666	0.855	0.937	0.968	**2.13**	**2.63**	**3.02**	**3.30**
		5	**3.84**	**8.63**	**12.43**	**15.65**	0.676	0.861	**0.939**	**0.969**	**2.13**	**2.63**	**3.02**	**3.30**

**Table 2:** Shopping mall noise (DCASE). Best performances in each category are marked with bold font.

Metric	*L*	*O*	SI-SDR (dB)				STOI				PESQ			
SNR			−5 dB	0 dB	5 dB	10 dB	−5 dB	0 dB	5 dB	10 dB	−5 dB	0 dB	5 dB	10 dB
original	-	-	−5.31	−0.31	4.68	9.53	0.471	0.638	0.776	0.879	1.70	2.02	2.36	2.74
Dilated	1	1	5.65	9.62	13.00	15.94	0.749	0.882	0.944	0.970	2.32	2.70	3.01	3.28
U-Net	5	1	5.31	9.34	12.72	15.52	0.735	0.871	0.939	0.968	2.20	2.58	2.91	3.21
		2	6.12	9.95	13.28	16.32	0.788	0.899	0.950	0.974	2.29	2.66	2.98	3.26
		3	6.41	10.13	13.44	16.51	0.812	0.908	0.953	0.975	2.34	2.70	3.01	3.28
		4	6.53	10.19	13.48	16.55	0.820	0.910	**0.954**	**0.976**	2.36	2.71	3.01	3.28
		5	6.57	10.21	13.48	16.56	**0.822**	**0.911**	**0.954**	**0.976**	2.36	2.71	3.01	3.28
Res	1	1	5.36	8.99	12.18	15.02	0.778	0.890	0.942	0.968	2.27	2.63	2.95	3.23
BLSTM	5	1	5.32	9.08	12.31	15.11	0.761	0.885	0.942	0.969	2.29	2.62	2.92	3.20
		2	5.86	9.48	12.65	15.49	0.802	0.902	0.948	0.971	2.39	2.71	2.99	3.24
		3	6.01	9.57	12.71	15.56	0.812	0.905	0.949	0.971	**2.40**	2.72	2.99	3.24
		4	6.03	9.59	12.72	15.56	0.815	0.906	0.949	0.971	**2.40**	2.72	2.99	3.24
		5	6.03	9.59	12.72	15.57	0.816	0.906	0.949	0.971	**2.40**	2.71	2.98	3.23
Trans-	1	1	5.42	9.46	12.94	16.05	0.750	0.881	0.943	0.971	2.16	2.56	2.92	3.24
former	5	1	4.96	9.24	12.78	15.75	0.714	0.861	0.936	0.969	2.16	2.55	2.92	3.25
-Net		2	6.06	9.98	13.42	16.59	0.777	0.895	0.950	0.975	2.30	2.67	3.01	3.31
		3	6.42	10.19	13.55	16.72	0.798	0.904	0.953	**0.976**	2.36	2.72	3.04	3.32
		4	6.55	10.25	13.58	16.74	0.804	0.907	0.953	**0.976**	2.37	**2.73**	**3.05**	**3.33**
		5	**6.59**	**10.26**	**13.60**	**16.75**	0.806	0.907	**0.954**	**0.976**	2.38	**2.73**	**3.05**	**3.33**

## Data Availability

All datasets used in this study are available (free or commercially). The scripts which we used to generate noise samples used for model training are available at https://drive.google.com/drive/folders/1KgOjTTcFfglBeSJSBM72WJY_cdek2Qmh?usp%3Dsharing&sa=D&source=docs&ust=1646776164896590&usg=AOvVaw2nK4omJ5lIZJ90m_9Ufd9H, accessed on 17 February 2022.
